# Five‐Year Serial Brain MRI Analysis of Military Members Exposed to Chronic Sub‐Concussive Overpressures

**DOI:** 10.1002/jmri.29419

**Published:** 2024-05-18

**Authors:** Rafael Glikstein, Gerd Melkus, Eduardo Portela de Oliveira, Maria Lucia Brun‐Vergara, Betty Anne Schwarz, Tim Ramsay, Tinghua Zhang, Christopher Skinner

**Affiliations:** ^1^ The Ottawa Hospital – Civic Campus Ottawa Ontario Canada; ^2^ Ottawa Hospital Research Institute, Ottawa Methods Centre Ottawa Ontario Canada

**Keywords:** MRI, cavernoma, developmental venous anomaly (DVA), brain volume loss, chronic traumatic encephalopathy

## Abstract

**Background:**

The Canadian Special Operations Forces Command conducts explosives operations and training which exposes members to explosive charges at close proximity. This 5‐year longitudinal trial was conducted in follow‐up to our initial trial which examined military breachers with MRI and EEG pre and post blast exposure.

**Purpose:**

To examine brain MRI findings in military personnel exposed to multiple repeated blast exposures.

**Study Type:**

Five‐year longitudinal prospective trial.

**Population:**

Ninety‐two males aged 23–42 with an average of 9.4 years of blast exposure.

**Field Strength/Sequence:**

3 T brain MRI/T1‐weighted 3D with reconstruction in three planes, T2‐weighted, T2‐weighted fluid attenuated inversion recovery (FLAIR) 3D with reconstruction in three planes, T2‐weighted gradient spin echo (GRE), saturation weighted images, DWI and ADC maps, diffusion tensor imaging.

**Assessment:**

All MRI scans were interpreted by the two neuroradiologists and one neuroradiology Fellow in a blinded fashion using a customized neuroradiology reporting form.

**Statistical Tests:**

Matching parametric statistics represented the number of participants whose brain parameters improved or deteriorated over time. Odds ratio (OR) and 95% confidence intervals (CI) were computed using log regression modeling to determine volume loss, white matter lesions, hemosiderosis, gliosis, cystic changes and enlarged Virchow Robin (VR) spaces. A Kappa (*κ*) statistic with a 95% CI was calculated to determine rater variability between readers.

**Results:**

A significant deterioration was observed in volume loss (OR = 1.083, 95% CI 0.678–1.731, permutation test), white matter changes (OR: 0.754, 95% CI 0.442–1.284, permutation test), and enlargement of VR spaces (OR: 0.775, 95% CI 0.513–1.171). Interrater reliability was low: *κ* = 0.283, 0.156, and 0.557 for volume loss, white matter changes, and enlargement of VR spaces, respectively.

**Data Conclusion:**

There were significant changes in brain volume, white matter lesions, and enlargement of VR spaces.

**Evidence Level:**

2

**Technical Efficacy:**

Stage 2

Primary blast injury is caused by the overpressure blast wave produced by high‐order explosives, primarily affecting air‐filled organs and cavities such as the ear, lung, and abdomen, as well as the head and neck. This blast wave is magnified by surface reflections, especially in confined spaces like buildings, buses, and trains.

During military activities such as breaching, and the use of high impulse weapons such as anti‐tank weapons, the brain can be exposed to considerable overpressures even when safeguarded by the current personal protective equipment in use.^1^

There is evidence that exposure to repeated blast overpressure is causing injury among personnel conducting explosive breaching in both training and operations.^2^ Breachers are military members who use explosive charges to enter secured buildings or take down armed threats. The nature and mechanism of these injuries is not well understood, nor is the amount and levels of blast overpressure that the Canadian Department of National Defence (DND) personnel are exposed to. However, the observed injury pattern resembles other forms of mild traumatic brain injury (mTBI). Radiological diagnosis of secondary blast injury helps to prioritize treatment by identifying life‐threatening conditions that may require timely intervention.

Radiological diagnosis of primary blast injury typically focuses on pulmonary (lung) and enteric (intestine) barotraumas, which involve damage to the lung or intestine from rapid or excessive pressure changes. In contrast, secondary blast injury is characterized by trauma resulting from the impact of bomb fragments, including the bomb casing and additional objects intentionally added for increase lethality (eg, screws, nails, nuts, and bolts). It can also result from external debris propelled by the explosion. Secondary blast injury primarily causes penetrating trauma but may also cause blunt trauma. Patients may experience penetration in various regions of the body, typically with fragment penetrations in multiple areas. Chronic traumatic encephalopathy (CTE) is a neurodegenerative disorder associated with exposure to repetitive head impacts, such as those sustained during contact and impact sports. It is important for clinicians, researchers, and the public to be aware that a definite diagnosis of CTE cannot be confirmed during a person's lifetime, and that individuals suspected of being at risk of CTE must be appropriately screened for potentially treatable conditions and associated comorbidities that can worsen or accelerate neurodegeneration.^3^

As time is essential in the clinical management of blast injury, clinicians should become familiar with the pathophysiology, diagnosis, and treatment considerations for TBI in advance of any explosive event. The brain is clearly vulnerable to both secondary blast injury (caused by flying debris and fragments) and tertiary blast injury (caused by being thrown by blast wind), involving penetrating and blunt trauma similar to head injuries from non‐explosive causes.

It has become a concern that sub‐acute effects of repeated blast exposure could still lead to chronic brain damage.^4^ The risk of long‐term neurological impairment from repetitive head impact may lead to a progressive neurodegenerative condition that may result in the development of CTE.^5^ Without proper surveillance of individuals exposed to repetitive head impact or blast injury, there is a lack of biomarkers to identify and track structural brain changes over time.^6^ Similarly, a study previously conducted involving retired Canadian Football League players who had suffered concussions from contact sports, matched with controls of similar age and education, was validated with controls from the Canadian Centre for Aging and Neuroscience database. This research found evidence of cognitive deficits, psychiatric impairment, and CTE.^7^

In a sample of 202 deceased American football players from the Brain Donation Program, 177 players of all skill levels (87%), including 110 out of 111 former NFL players (99%), were neuropathologically diagnosed as CTE. In addition, a large proportion showed pathological signs indicative of CTE, suggesting an association between CTE and previous participation in football. American football players may face an elevated risk of long‐term neurological diseases, particularly CTE.^8^ Additionally, it has also been reported that CTE‐related lesions are associated with single, moderate‐to‐severe TBI events.^5^ Finally, in a recent publication examining the longitudinal changes in regional brain volumes and cognition in professional fighters with traumatic encephalopathy syndrome was validated with a control group.^9^

It is estimated that more than 1.75 million US citizens per year suffer a TBI, whereby 75%–85% are categorized as mild (mTBI).^10^, ^11^ Considering that many other cases are seen in private clinics and 25% of individuals seek no medical attention, the yearly incidence of mTBI is estimated to be higher. Given the large numbers of known and unlikely known cases, TBI has been coined as the “silent epidemic.” Globally, long‐term cognitive deficits from TBI‐related injuries in sport events have been broadly reported. In addition, soldiers returning from Iraq and Afghanistan frequently report TBI from combat‐related injuries.^12^ The frequency of these reports has resulted in TBI being referred to as “signature injury of war.” Soldiers arriving at Landstuhl Regional Medical Centre in Germany with facial and neck injuries also suffered TBI due to exposure to improvised explosive devices (IEDs), landmines, high pressure waves from blasts, head injury from blunt force trauma, and motor vehicle accidents. It is especially important to note that in 2008, all service members who returned from Iraq and Afghanistan; (300,000 service members in total) sustained at least one incidence of mTBI. Therefore, mTBI is a major health concern that impacts both civilian and military population, resulting in 44% of the economic costs of 56 billion dollars spent annually treating TBI in the United States.^13^

In an effort to mitigate such injuries, the DND commissioned this study to explore the impact of blast over pressure exposure on the brain structure of its personnel. To that end, in partnership with a tertiary medical center, DND sponsored this longitudinal study aimed to track and record the effects of blast overpressure exposure within the operator community over a 5‐year period. Thus, the aim of this study was to investigate the comprehensive impact of cumulative blast operations and trainings on brain structure over a 5‐year period.

## Material and Methods

The project was approved by the local regulatory ethics board and each subject gave written informed consent, after being fully apprised of the purpose of the 5‐year longitudinal study with its risks and benefits. Inclusion criteria included: military members actively involved in explosives operations or training, in good health with no reported previous neurological problems. However, there was radiological evidence of brain malformation, including brain volume loss, WM lesions, and Virchow‐Robin space enlargement because of exposure to frequent sub concussive overpressure.

Members with a history of claustrophobia and those who did not sign consent were excluded.

All MRI scans were performed at a tertiary medical center on a 3 T MRI scanner (Magnetom Trio, Siemens Healthineers, Erlangen, Germany) using a 32‐channel receive only head coil. Analysis of these MRI scans was done to assess variation in gray and white matter concentration as a function of repetitive blast exposure. The complete MRI protocol and parameters are listed in Table 1.

**TABLE 1:** MRI Protocol and Parameters

Sequence	T1‐Weighted GRE	T2‐Weighted FLAIR	T2‐Weighted TSE	DTI EPI	DWIEPI	T2*‐Weighted EPI FID	SWI GRE
Orientation	Axial	Sagittal	Axial	Axial	Axial	Axial	Axial
Type	3D	3D	2D	2D	2D	2D	3D
Fat supression	Yes	No	Yes	Yes	Yes	Yes	No
TR [msec]	8.5	5000	6730	9100	5300	7080	28
TE [msec]	3.2	441	99	70	79	59	20
Tl [msec]	n.a.	1800	n.a.	n.a.	n.a.	n.a.	n.a.
Flip angle [degree]	12	T2‐var	90, 120	90, 180	90, 180	45	15
FOV [mm × mm]	256 × 256	256 × 256	220 × 180	192 × 192	230 × 230	230 × 230	230 × 172
Matrix	256 × 256	256 × 256	320 × 256	128 × 128	160 × 160	214 × 214	384 × 288
Resolution [mm × mm]	1 × 1	1 × 1	0.7 × 0.7	1.5 × 1.5	1.4 × 1.4	1.1 × 1.1	0.6 × 0.6
Slice thickness [mm]	1	1	3	1.5	3	3	1.8
Number of slices	208	160	40	66	40	40	80
Parallel imaging factor	2	2	2	2	2	2	2
Bandwidth/pixel [Hz/pixel]	130	781	300	1860	1488	1230	120
*b*‐values [s/mm^2^]	n.a.	n.a.	n.a.	0, 1000 (20 directions)	0, 500, 1000 (trace)	n.a.	n.a.
Averages	1	1	1	1	1	3	1
Total time	2 min 37 s	5 min 57 s	1 min 22 s	3 min 40 s	55 s	28 s	6 min 14 s

The intent was that each participant would have a baseline MRI scan and then follow‐up scans over the next 5 years. It was deemed important to perform screening of individuals prior to exposure to blast exposure to rule out structural lesions which may have deleterious effects affecting the member's health if exposed to upon repeated exposure to blast injury. The MRI protocol was designed to detect various conditions, including detection of acute ischemic injury, detection of white matter lesions, detection of hemorrhagic lesions, detection of damage to white matter tracts because of diffuse axonal injury, detection of other interval changes that may not manifest with clinical symptoms, such as subdural hemorrhages or fluid collection, and to detect volumetric changes over time.

The MRI protocols included the following modality sequences: T1‐weighted 3D with reconstruction in three planes, T2‐weighted, T2‐weighted fluid attenuated inversion recovery (FLAIR) 3D with reconstruction in three planes, T2‐weighted gradient spin echo (GRE), susceptibility weighted images (SWI), diffusion weighted imaging (DWI), and apparent diffusion coefficient (ADC) maps. The T1 and T2 datasets were used to detect any hemorrhagic injury caused by blast exposure. The FLAIR, DWI, and ADC maps were used to determine any acute ischemic injury caused by blast exposure. The DTI was used to determine damage to white matter tracts because of blast exposure.

All MRI scans were interpreted by two neuroradiologists RG Reader 1 (30 years), EPO Reader 2 (8 years), and one neuroradiology Fellow MLBV Reader 3 (2.5 years' experience) in a blinded fashion using a customized neuroradiology reporting form (see Figure S1). Rater reliability was calculated with Kappa κ statistics (Table 4).

This procedure facilitated the objective quantification of structural changes in the brain based on location and MRI protocol. All anonymized MRI scans were interpreted by year and not sequentially to reduce any risk of bias from recency effect. This procedure facilitated the objective quantification of structural changes in the brain based on location and MRI protocol. Specific findings were documented and codified in the reporting sheet including volume loss, white matter changes, gliosis, the presence of hemosiderin deposits, and expansion of VR spaces by location the radiology reporting form also provided space for text entry of incidental findings. Any incidental MRI findings were reported to the principal investigator for remedial action through the subject's primary health care provider. The results of the reporting sheets were then recorded into a database for future analysis and comparison for changes over time (Table 5, Table 7).

### Statistical Analysis

All statistical analyses were performed using SAS. Version 9.4 (SAS Institute Inc. Cary, North Carolina, USA). To evaluate the statistical significance of the deterioration rate, a permutation test was conducted. In this test, the order of each participant's scans was randomly permuted, and the deterioration rate was calculated for each permutation. This permutation procedure was repeated 9999 times, with each deterioration rate recorded and these rates were combined with the observed (unpermuted) deterioration rate. Under the null hypothesis that there was no true deterioration, the *P*‐value was defined as the probability of observing a deterioration rate as large or larger than the observed deterioration rate between the first and last MRI scan (Table 3).

Logistic regression models were employed, with the outcome variables including volume loss, white matter lesions, hemosiderosis, gliosis, cystic changes, VR spaces from last scan and predictors as age and years of exposure. The adjusted odds ratios are presented with 95% Cl and *P*‐value. The permutation tests were one‐sided at the 5% significance level, while all other tests were two sided at the 5% level. Kappa with a CI of 95% was calculated to measure rater reliability between MRI readers.

## Results

The study population consisted of 213 MRI scans from 92 subjects. The population was all male with an age range from 23 to 42 years; with an average age of 31, and an average of 9.4 years of blast exposure.

There were seven drops‐outs in the trial, which was a small occurrence related to the subject's availability or changes in career options. There were several incidental findings requiring remedial medical follow‐up. None of these individuals withdrew from the study.

During the study, some members were either no longer a breacher with the military or they were deployed and not able to return on an annual basis for their MRI brain. Table 2 depicts the follow‐up study visits and MRI brain that occurred from 2016 to 2022.

There was a significant deterioration for volume loss (OR = 1.083, 95% CI 1.678–1.731, permutation test), white matter changes (OR 0.754, 95% CI 0.442–1.284, permutation test) and enlarged VR spaces (OR: 0.775, 95% CI 0.513–1.171).

**FIGURE 1:**
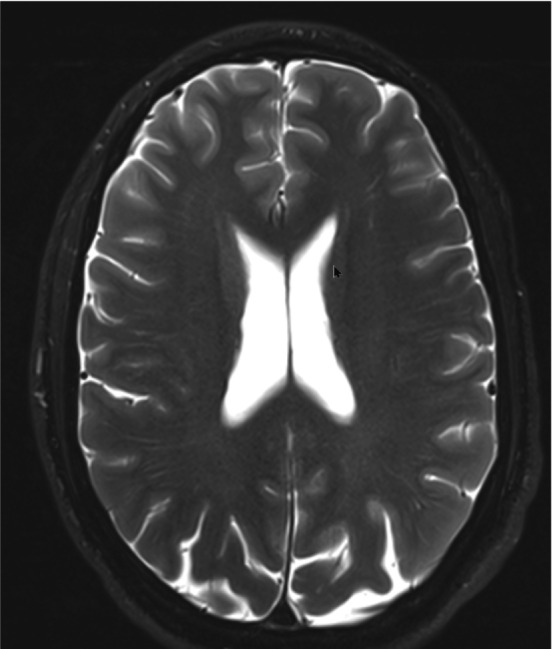
Example of enlarged VR spaces. T2 FLAIR image showing enlarged VR spaces mainly in the posterior quadrants.

**FIGURE 2:**
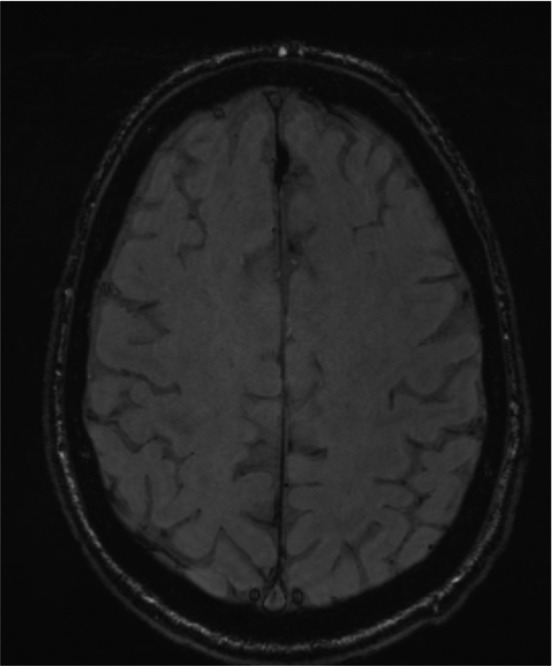
Example of hemosiderosis. SWI image showing superficial hemosiderin deposit.

**FIGURE 3:**
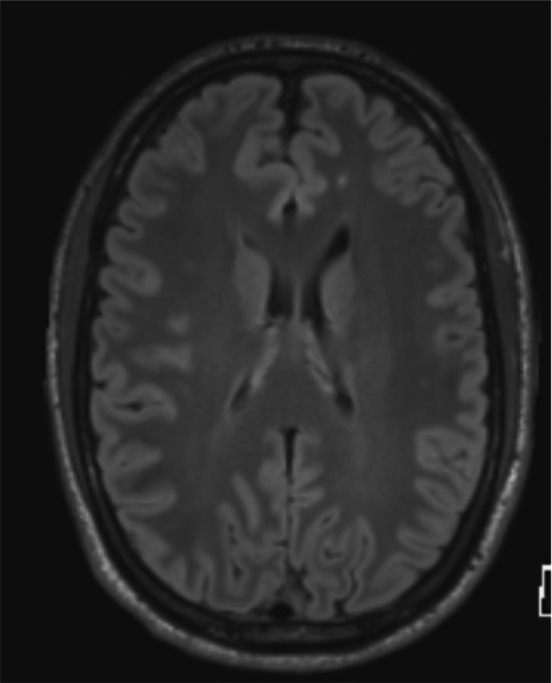
Example of white matter changes. T2 FLAIR image showing multifocal white matter changes.

An example of enlarged VR spaces (Figure 1) and evidence of excessive accumulation of iron known as hemosiderosis (Figure 2) on brain MRI were found. An example of multifocal white matter changes on T2 FLAIR was also found (Figure 3). No definite findings of new onset ischemic injury were observed in any of the subjects.

The log regression model analysis for the various criteria such as volume loss, gliosis, VR, cystic changes, white matter changes in relation to age and years of exposure were statistically significant (Appendix). Hemosiderin deposits was not statistically different. These included two cavernomas, one epidural cyst impinging on the pons, and 26% with developmental venous anomalies (DVAs) (Table 6). The study did not reveal any progression or new hemorrhages in these two individuals. Similarly, the subjects with DVAs showed no evidence of hemorrhage or changes in the vascular anatomy (Table 3).

**TABLE 2:** MRI Exams Completed and Number of Military Members No Longer With the Unit

Year	2016	2017	2018	2019	2020	2021	2022	Total
Scans	18	30	52	63	25	31	7	213
No longer with the unit	0	0	4	1	0	2	0	7

**TABLE 3:** Permutation Test

Brain Parameter	N	Imp.rate	Det.rate	*P*
VL	68	3%	15%	0.0001
WM	68	4%	13%	0.0001
HS	68	0%	1%	0.998
GL	68	1%	3%	0.0138
CY	68	1%	4%	0.0001
VR	68	6%	28%	0.0001

N represents the number of evaluable participants for each brain parameter, Imp.rate represents the percent showing improvement over time, Det.rate represents the percent showing deterioration, and *P* is the *P*‐value for the deterioration rate.

As improvement is most likely impossible; it may have been affected by radiologists' reliability. Det.rate represents the percent showing deterioration. *P* is the *P*‐value for the deterioration rate. Under the null hypothesis there was no true deterioration, the *P*‐value is defined as the probability of observing a deterioration rate as large or larger than the observed deterioration rate. The statistically significant level is usually set as 0.05. Only HS is not statistically significant. The remaining five parameters are statistically significant.

The study identified several individuals with vascular lesions that might have been influenced by repetitive exposure to blast as part of their training or employment. These included two cavernomas, one epidermoid cyst impinging on the pons and 26% with DVAs (Table 6). The study did not reveal any progression or new hemorrhages in these two individuals. Similarly, the subjects with DVAs showed no evidence of hemorrhage or changes in the vascular anatomy.

**TABLE 4:** Kappa Statistics Between Readers

Reader	Kappa	95% CI		*P*	Strength of Agreement
1 and 2	0.156	0.0481	0.2633	0.0015	Slight
1 and 3	0.283	0.1757	0.3894	<0.0001	Fair
2 and 3	0.557	0.4596	0.6544	<0.0001	Moderate

The following is general Kappa interpretation <0 poor, 0.0–0.20 slight, 0.21–0.4 fair, 0.41–0.6 moderate, 0.61–0.8 substantial, and 0.81–1 almost perfect.

## Discussion and Conclusion

Over the lifespan, the human brain undergoes microstructural changes, including gray matter atrophy, myelin degeneration, or iron accumulation, that can be detected in vivo by various quantitative MRI techniques. Understanding the extent of age‐related changes in brain structure and associated MR parameters is important for understanding the neurobiology of aging, and for distinguishing physiological aging from disease.^14^ Interestingly, the literature suggests that there is an age‐related increase in incidence of cavernomas in individuals with DVAs.^15^

Screening of individuals prior to exposure, particularly for employment in environments with repetitive blast exposure, to rule out structural lesion to avoid deleterious effects on the individual's health has not been extensively studied. The study identified several individuals with vascular lesions that might have been influenced by repetitive exposure to blast as part of their training or employment. With respect to analysis only members with completion of two or more MRI scans were evaluated for a total of 68 evaluable MRI scans. Due to logistical constraints in this military population, there was difficulty maintaining continuity of scans over the 5 years of the study.

In this study, a significant deterioration was observed in brain MRI for volume loss, white matter changes and enlarged VR spaces in military personnel exposed to multiple repeated blast exposure.

The permutation analysis supports the hypothesis that there were changes in brain volume, increased white matter lesions, and enlargement of VR space over the course of the study.

The log regression model analysis for the various criteria such as volume loss, gliosis, VR, cystic changes, white matter changes, in relation to age and years of exposure were statistically significant. Hemosiderin deposits was not statistically significant.

The finding of brain volume loss requires correlation with other objective measures, such as biomarkers. In addition, the incidental discovery of two cavernomas in this cohort highlights the need for pre‐selection screening using high resolution MRI scanning. There is limited literature on the life history of individuals in such environments with cavernomas.

The issue of DVAs is more problematic, as the incidences in the normal population of this age is 9.6%,^16^ and raises the question whether these vascular abnormalities represent a risk for progression or rupture in this population. It is unclear if this increased incidence is related to exposure or influenced by sampling bias.

This study did not find any changes or complications related to the DVAs over the course of the study.

Of most concern is whether the findings of decreased brain volume, increased white matter lesions and enlargement of Virchow Robin spaces changes represent a neurodegenerative process, potentially placing these individuals on the spectrum of CTE.

**TABLE 5:** MRI Summary of Findings

Summary of Findings	
Volume loss	62%
White matter changes	66%
Hemosiderin	4%
Gliosis	6%
Cystic changes	7%
Virchow Robin spaces	53%

**TABLE 6:** MRI Summary of Incidental Findings

Summary of Incidental Findings	
Sinusitis	55%
Developmental venous anomaly	26%
Asymmetry	18%
Gliosis	6%
Polyps	9%
Cavernoma	2%
Epidermoid cyst	1%

**TABLE 7:** Summary of Radiological Findings

Categories	2016	2017	2018	2019	2020	2021	2022
Volume loss	48	19	35	41	17	12	2
White matter changes	41	32	40	40	23	17	3
Hemosiderin	0	0	3	5	1	0	3
Gliosis	4	1	8	4	0	0	0
Cystic changes	1	1	6	7	3	2	1
Virchow Robin spaces	31	22	27	35	21	21	1

### Limitations

The sample for this trial was small and lacked a control group, and thus does not provide strong evidence of a direct causal link between blast exposure and MRI changes.

However, as previously reported control groups exposed to concussions from contact sports found evidence of cognitive and psychiatric symptoms plus CTE. It would have been beneficial to conduct neuropsychological assessments annually to measure if there were cognitive impairments to correlate with the MRI findings.

In addition, the incidental discovery of two cavernomas in this cohort highlights the need for pre‐selection screening using high resolution MRI scanning. The incidence of DVAs raises the question whether these vascular abnormalities represent a risk for progression or rupture in this population. It is unclear whether pre‐existing DVAs pose any risk to the individual related to blast exposure. This study did not find any changes or complications related to the DVAs over the course of the study.

Of most concern is whether the findings of decreased brain volume, increased white matter lesions and enlargement of Virchow Robin spaces changes represent a neurodegenerative process, potentially placing these individuals on the spectrum of CTE.

This study demonstrated significant alterations in brain volume, increased white matter lesions, and enlargement of VR spaces over the study duration in a normal fit male population of physically fit male military members.

## Supporting information


**Figure S1:** Customized neuroradiology reporting form.

## Appendix 1 Log Regression Model Analysis

**Table:**

Fit Logistic Regression Models for Each Outcome as Last Scan of VL, WM, HS, GL, CY, VR				
Probability Modeled is VL = 1 (Volume Loss)				
	OR	95% CI		*P*
Age	0.983	0.621	1.555	0.9401
Years_of_Exposure	1.083	0.678	1.731	0.7385
N = 68				

Probability Modeled is WM = 1 (White Matter Lesions)				
	OR	95% CI		*P*
Age	1.514	0.884	2.595	0.1309
Years_of_Exposure	0.754	0.442	1.284	0.2986
N = 68				

Probability Modeled is HS = 1 (Hemosiderosis)				
	OR	95% CI		*P*
Age	2.492	0.704	8.824	0.1569
Years_of_Exposure	0.393	0.11	1.401	0.1501
N = 68				

Probability Modeled is GL = 1 (Gliosis)				
	OR	95% CI		*P*
Age	1.197	0.445	3.222	0.7216
Years_of_Exposure	0.978	0.353	2.708	0.9654
N = 68				

Probability Modeled is CY = 1 (Cystic Changes)				
	OR	95% CI		*P*
Age	0.695	0.286	1.687	0.4212
Years_of_Exposure	1.818	0.697	4.741	0.2215
N = 68				

Probability Modeled is VR = 1 (VR Spaces)				
	OR	95% CI		*P*
Age	1.52	0.995	2.323	0.053
Years_of_Exposure	0.775	0.513	1.171	0.2265
N = 68				
