# Supplementary material for: Five‐Year Serial Brain MRI Analysis of Military Members Exposed to Chronic Sub‐Concussive Overpressures
Source: J Magn Reson Imaging. 2024 May 18;61(1):415–23. doi: 10.1002/jmri.29419 (PMC11645495; doi:10.1002/jmri.29419)
Supplement: Supplementary file 1 — Figure S1: Customized neuroradiology reporting form. [file JMRI-61-415-s001.docx]

| **ZAP:** | **Right Tl** | **Left Tl** | **Right T2** | **Left T2** | **Right T2 Flair** | **Left T2 Flair** | **Right SWI** | **Left SWI** | **Right GRE** | **Left GRE** | **Right DTI** | **Left DTI** | **COMMENTS** |
| --- | --- | --- | --- | --- | --- | --- | --- | --- | --- | --- | --- | --- | --- |
| **Date of Scan:** |  |  |  |  |  |  |  |  |  |  |  |  |  |
| **EXPOSURE: Y or N** |  |  |  |  |  |  |  |  |  |  |  |  |  |
| **Serial Number:** |  |  |  |  |  |  |  |  |  |  |  |  |  |
| **Neuroradiologist:** |  |  |  |  |  |  |  |  |  |  |  |  |  |
| **Date Interpreted:** |  |  |  |  |  |  |  |  |  |  |  |  |  |
| **Gliosis** |  |  |  |  |  |  |  |  |  |  |  |  |  |
| **Cystic Changes** |  |  |  |  |  |  |  |  |  |  |  |  |  |
| **White volume loss** |  |  |  |  |  |  |  |  |  |  |  |  |  |
| **No volume loss** |  |  |  |  |  |  |  |  |  |  |  |  |  |
| **Mild volume loss** |  |  |  |  |  |  |  |  |  |  |  |  |  |
| **Moderate volume loss** |  |  |  |  |  |  |  |  |  |  |  |  |  |
| **Severe volume loss** |  |  |  |  |  |  |  |  |  |  |  |  |  |
|  |  |  |  |  |  |  |  |  |  |  |  |  |  |
|  |  |  |  |  |  |  |  |  |  |  |  |  |  |
| **Caudate** |  |  |  |  |  |  |  |  |  |  |  |  |  |
| **Putamen** |  |  |  |  |  |  |  |  |  |  |  |  |  |
| **Globus Pallidus** |  |  |  |  |  |  |  |  |  |  |  |  |  |
| **Thalamus** |  |  |  |  |  |  |  |  |  |  |  |  |  |
|  |  |  |  |  |  |  |  |  |  |  |  |  |  |
| **External Capsule** |  |  |  |  |  |  |  |  |  |  |  |  |  |
| **Internal Capsule Anterior Limb** |  |  |  |  |  |  |  |  |  |  |  |  |  |
| **Internal Capsule Anterior Limb** |  |  |  |  |  |  |  |  |  |  |  |  |  |
| **Pyridimal** |  |  |  |  |  |  |  |  |  |  |  |  |  |
| **Brain stem** |  |  |  |  |  |  |  |  |  |  |  |  |  |
|  |  |  |  |  |  |  |  |  |  |  |  |  |  |
| **Legend** |  |  |  |  |  |  | **Degree of Severity** | |  |  |  |  |  |
|  | | | | | **none** | **1-20%** | **20-40%** | **40-60%** | **60-80%** | **80-100%** |  |  |  |
|  |  |  |  |  | **0** | **1** | **2** | **3** | **4** | **5** |  |  |  |

Please check rate of severity as per legend provided.

Abbreviations

WM – white matter, SC – subcortical

FLAIR – Fluid-Attenuated Inversion Recovery, T2-weighted

VR – Virchow Robin space
